# Adult ileo-ileal intussusception caused by inflammatory fibroid polyp leading to small bowel obstruction: a case report with comprehensive literature review

**DOI:** 10.1093/jscr/rjae480

**Published:** 2024-08-03

**Authors:** Shritik Devkota, Hritika Rathi, Arshid Iqbal Qadri, Samiksha Lamichhane, Nidhi Jain

**Affiliations:** Department of Radiodiagnosis & Imaging, Anil Baghi Hospital, Punjab, 152002, India; Department of Radiodiagnosis & Imaging, Postgraduate Institute of Medical Education and Research, Chandigarh, 160012, India; Department of Radiodiagnosis & Imaging, Postgraduate Institute of Medical Education and Research, Chandigarh, 160012, India; Department of Surgical Gastroenterology, Anil Baghi Hospital, Punjab, 152002, India; Department of Radiodiagnosis & Imaging, B. P. Koirala Institute of Health Sciences, Dharan, 56700, Nepal; Department of Pathology, Anil Baghi Hospital, Punjab, 152002, India

**Keywords:** adult ileo-ileal intussusception, small bowel obstruction, inflammatory fibroid polyp, small bowel obstruction, computed tomography in adult ileo-ileal intussusception

## Abstract

Ileo-ileal intussusception, an infrequent cause of small bowel obstruction in adults, can be initiated by inflammatory fibroid polyps. These are uncommon, benign submucosal lesions of the gastrointestinal tract. This case report explores an adult patient with inflammatory fibroid polyps-induced ileo-ileal intussusception.

## Introduction

Intussusception occurs when a segment of intestine (intussusceptum) telescopes into the adjacent segment (intussuscipiens) along with its mesenteric fold [1]. This telescoping, also known as invagination, can arise from various causes. In the small bowel, benign growths like lipomas and leiomyomas are frequent culprits. Inflammatory fibroid polyps (IFPs), while less common, can also be a cause. Conversely, malignant lesions are more often implicated in large intestine intussusception [2]. Notably, IFPs are typically benign submucosal tumors residing in the stomach, although they can occur throughout the gastrointestinal tract [3, 4].

Imaging plays a pivotal role in diagnosing intussusception. Various techniques, including plain abdominal radiography, ultrasound, barium contrast studies, computed tomography (CT), and magnetic resonance imaging (MRI) are employed [5–7]. Ultrasound often reveals a characteristic ‘target-like’ or ‘bull’s-eye’ appearance. CT can identify the causative lesion and characteristic signs like the ‘bowel-within-bowel’ configuration, potentially containing fat and mesenteric vessels [5–7]. Definitive diagnosis of a lead point, such as an IFP, requires histopathological assessment coupled with immunohistochemistry [2, 7, 8]. Given the increased risk of malignancy in adults, surgery is the mainstay of treatment [5, 7].

This case report presents a rare occurrence of ileo-ileal intussusception triggered by an IFP in a 35-year-old woman with intestinal obstruction symptoms. A combination of CT scan, intraoperative findings, and histopathological analysis established the final diagnosis, excluding other possibilities.

## Case report

A 35-year-old woman presented to the emergency department with a 3-day history of lower abdominal pain, constipation, and a 2-day fever. She also reported six episodes of vomiting. Notably, she was able to pass flattus. The patient denied any prior episodes of similar symptoms or any significant past surgical or medical history. Her vitals including pulse and blood pressure were within normal limits. There were no physical signs of dehydration. Examination revealed localized tenderness on deep palpation at hypogastrium, however, there was no rebound tenderness. Her basic blood work up including complete blood count, renal and liver function tests were within normal limits.

**Figure 1:**
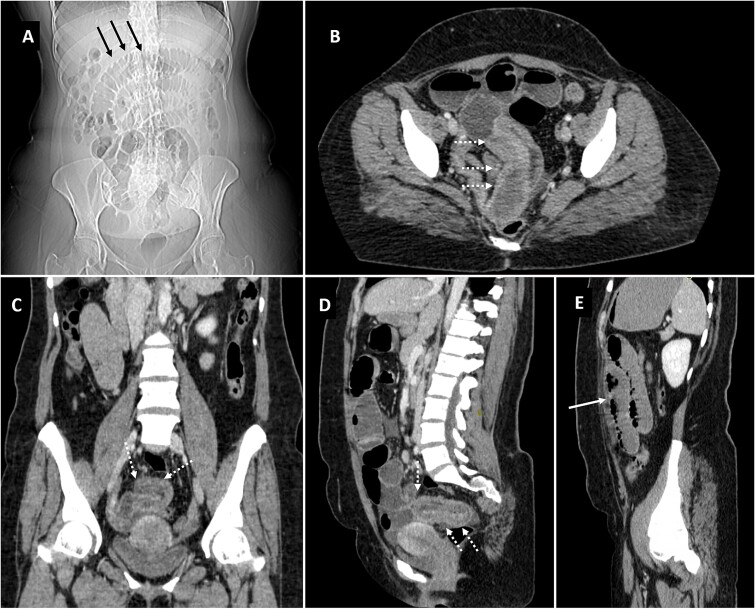
Scannogram (A) showing dilated small bowel loops in central abdomen (black arrows). Axial (B), coronal (C) and sagittal (D, E) images of contrast CT showing telescoping of pelvic ileal loop (dotted white arrows) with upstream small bowel obstruction (solid white arrow).

The patient was shifted to the radiology department for screening ultrasonography which revealed dilated small bowel loops with to-and-fro peristalsis with maximum caliber of 3.5 mm. With provisional diagnosis of intestinal obstruction, patient was planned for CT scan to localize the transition point. CT scan (Fig. 1) demonstrated telescoping of one of the pelvic ileal loops into adjacent ileal loop for a length of ~14 cm. The distal bowel segment receiving the prolapsing bowel segment showed circumferential mural thickening measuring approximately 7 mm with resultant dilatation of the proximal jejunal, and mid ileal pelvic loops which showed air fluid levels measuring upto 3.6 cm in maximum caliber. The distal bowel loops beyond this point appeared collapsed. Thus, a diagnosis of ileo-ileal intussuception was established.

The patient was informed about the diagnosis and the need for surgery. Surgical resection of the intussuscepted segment was performed, followed by anastomosis of the adjacent ileal segments. There were no complications during surgery or the immediate postoperative period. Oral fluids were gradually introduced after 3 days, followed by a transition to solid foods after 5 days. The patient made a good recovery. A follow-up appointment 2 weeks later confirmed good healing of the surgical incision.

**Figure 2:**
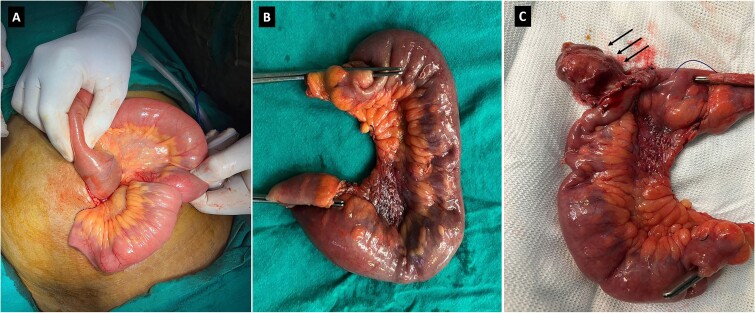
Intraoperative image showing the small bowel segment with ileo-ileal intussusception (A). Surgical specimen (B) showing the resected ileal segment. Surgical specimen (C) showing polypoid lesion (black arrows).

**Figure 3:**
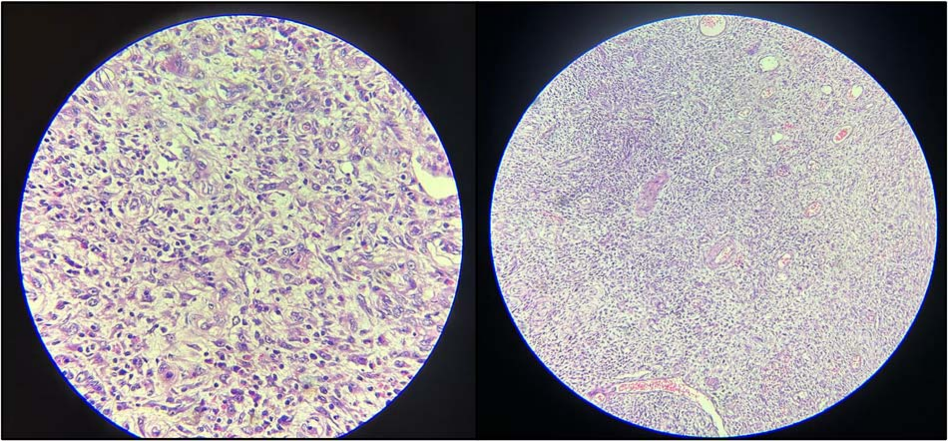
Histopathological examination reveals spindle-shaped cells within a loose, fibromyxoid background. The infiltrate of inflammatory cells, predominantly eosinophils and the presence of variably sized blood vessels are also evident.

The gross examination of the resected specimen revealed a 15 × 4 cm segment of intestine containing a submucosal polypoid lesion measuring 2 × 2 × 1 cm, located 4 cm from one end and 10 cm from the other (Fig. 2). No lymph nodes were identified in the specimen. Microscopic examination (Fig. 3) demonstrated a submucosal tumor composed of spindle-shaped cells within a loose fibromyxoid background. There was an infiltrate of inflammatory cells, predominantly eosinophils, and variably sized blood vessels. Based on these findings, the diagnosis was suggestive of an inflammatory fibroid polyp. Importantly, the proximal and distal resection margins were free of tumor involvement.

## Discussion

Intussusception arises when a segment of intestine telescopes into the adjacent lumen, along with its associated mesentery. The precise etiology remains elusive, although theories suggest that intraluminal irritants disrupt normal peristalsis, triggering the process [2]. Untreated intussusception can progress to ischemia, necrosis, and perforation [5, 7, 9, 10].

Adult intussusception is a rare entity, with a global annual incidence of ~1 in 1 000 000 [9]. Unlike their pediatric counterparts, which frequently lack a clear culprit, ~ 90% of adult cases harbor an identifiable lead point. Clinical presentation is variable, often encompassing a constellation of symptoms such as abdominal pain, nausea, vomiting, and constipation. Less frequent manifestations include melena, weight loss, and fever [5, 7, 9, 10]. The small intestine is the most common location for adult intussusception, with a predominantly benign underlying etiology [ 5, 7, 9, 10]. Potential causes encompass a spectrum of conditions, including strictures, adhesions, foreign bodies, vascular abnormalities, trauma, celiac disease, cytomegalovirus colitis, lupus-associated lymphoid hyperplasia, Meckel’s diverticulum, and non-malignant growths like lipomas and leiomyomas. IFPs represent another, less frequent, causative factor [3–7].

Compared to pediatric cases, adult intussusception of the large bowel exhibits a stronger association with malignant etiologies. Primary neoplasms, including adenocarcinoma and lymphoma, are frequently implicated, alongside secondary involvement from metastatic disease. Additionally, carcinoids, leiomyosarcomas, and gastrointestinal stromal tumors can also contribute to this phenomenon [3–7, 9, 10].

First described in 1949 by Vanek as ‘gastric submucosal granuloma with eosinophilic infiltration,’ IFP is a rare, benign submucosal mesenchymal neoplasm of the gastrointestinal tract. Predominantly affecting the stomach in older adults, with a female predilection, IFP can also manifest in the small intestine [2, 4, 11, 12]. Histologically, IFPs display haphazardly arranged stellate/spindle/epithelioid cells in myxoid/edematous stroma with prominent eosinophils and small vasculature. Immunoprofile is variable; positive for vimentin and CD34, negative for SMA, ALK1, CD117, S100, beta-catenin, and desmin [1–4, 8, 13].

Accurate diagnosis hinges on a meticulous medical history, thorough clinical examination, and a tailored selection of imaging modalities. These may include plain radiography, ultrasound, barium contrast studies, CT, and MRI. Plain abdominal X-ray often serves as the initial imaging tool, but typically reveals nonspecific air-fluid levels suggestive of obstruction [2, 5, 10]. Sonographic characteristics include the appearance of a target or doughnut sign in the transverse view, and in longitudinal view, it may display a pseudo-kidney, sandwich, or hayfork sign [5, 7, 10].

CT surpasses ultrasound for intussusception diagnosis due to its unimpeded visualization by gas. It boasts high sensitivity (58%–100%) [1] and aids in evaluation of location and extent of the lesion or lead point proximity to surrounding structures and potential complications [3, 6, 7, 10, 14]. CT scan effectively identifies ileo-ileal intussusception caused by benign polyps. These polyps appear as elongated, fat-containing, pedunculated filling defects within the proximal ileum [2, 6, 7, 14–16]. Additionally, CT demonstrates characteristic findings of small bowel obstruction, including proximal bowel dilation and a transition point to the distal, collapsed segment. A hallmark sign on CT is the ‘bulls-eye’ or ‘target’ appearance in axial sections and the ‘sausage-shaped’ lesion in coronal sections, collectively known as the double-ring sign [ 2, 6, 14–16].

MRI advancements, particularly the half-Fourier acquisition single-shot turbo spin-echo technique, are increasing diagnostic accuracy for bowel diseases, albeit with lower utilization compared to other modalities [1, 17, 18]. This technique offers a characteristic ‘bowel-within-bowel’ or ‘coiled-spring’ appearance on imaging. Additionally, a combination of T2-weighted and diffusion sequences with contrast-enhanced T1-weighted imaging can facilitate polyp detection [1, 17, 18].

In pediatric intussusception, pneumatic reduction under fluoroscopy or hydrostatic reduction under ultrasound guidance reigns supreme due to its high success rate and minimal perforation risk. Conversely, surgical intervention (laparotomy or laparoscopy) remains the mainstay in adults. This is driven by the frequent presence of an underlying pathology, such as malignancy, requiring resection and histopathological confirmation [1, 2, 5, 7, 10, 19].

## Conclusion

Adult intussusception, a rare entity, often presents with acute intestinal obstruction. Prompt and accurate diagnosis is crucial to prevent complications like bowel infarction and perforation, and to facilitate resection of the underlying lead point, particularly when malignancy is a potential culprit. Imaging plays a pivotal role in guiding diagnosis and treatment for these patients. Therefore, a thorough understanding of both the clinical presentation and the characteristic imaging findings of intussusception is paramount for optimal management and minimizing potential complications.

## Data Availability

Data sharing is not applicable to this article as no new data were created or analyzed.
